# Cryo-EM structure of the *Plasmodium falciparum* 80S ribosome bound to the anti-protozoan drug emetine

**DOI:** 10.7554/eLife.03080

**Published:** 2014-06-09

**Authors:** Wilson Wong, Xiao-chen Bai, Alan Brown, Israel S Fernandez, Eric Hanssen, Melanie Condron, Yan Hong Tan, Jake Baum, Sjors HW Scheres

**Affiliations:** 1 Division of Infection and Immunity, Walter and Eliza Hall Institute of Medical Research, Melbourne, Australia; 2 Department of Medical Biology, University of Melbourne, Melbourne, Australia; 3 Structural Studies, Medical Research Council Laboratory of Molecular Biology, Cambridge, United Kingdom; 4 Electron Microscopy Unit, Bio21 Molecular Science and Biotechnology Institute, University of Melbourne, Melbourne, Australia; Max Planck Institute of Biophysics, Germany

**Keywords:** malaria, *Plasmodium falciparum*, ribosome, drug development, cryo-EM, other

## Abstract

Malaria inflicts an enormous burden on global human health. The emergence of parasite resistance to front-line drugs has prompted a renewed focus on the repositioning of clinically approved drugs as potential anti-malarial therapies. Antibiotics that inhibit protein translation are promising candidates for repositioning. We have solved the cryo-EM structure of the cytoplasmic ribosome from the human malaria parasite, *Plasmodium falciparum*, in complex with emetine at 3.2 Å resolution. Emetine is an anti-protozoan drug used in the treatment of ameobiasis that also displays potent anti-malarial activity. Emetine interacts with the E-site of the ribosomal small subunit and shares a similar binding site with the antibiotic pactamycin, thereby delivering its therapeutic effect by blocking mRNA/tRNA translocation. As the first cryo-EM structure that visualizes an antibiotic bound to any ribosome at atomic resolution, this establishes cryo-EM as a powerful tool for screening and guiding the design of drugs that target parasite translation machinery.

**DOI:** http://dx.doi.org/10.7554/eLife.03080.001

## eLife digest

Each year, malaria kills more than 600,000 people, mostly children younger than 5 years old. Humans who have been bitten by mosquitoes infected with malaria-causing parasites become ill as the parasites rapidly multiply in blood cells. Although there are several drugs that are currently used to treat malaria, the parasites are rapidly developing resistance to them, setting off an urgent hunt for new malaria drugs.

Developing new antimalarial medications from scratch is likely to take decades—too long to combat the current public health threat posed by emerging strains of drug-resistant parasites. To speed up the process, scientists are investigating whether drugs developed for other illnesses may also act as therapies for malaria, either when used alone or in combination with existing malaria drugs.

Certain antibiotics—including one called emetine—have already shown promise as antimalarial drugs. These antibiotics prevent the parasites from multiplying by interfering with the ribosome—the part of a cell that builds new proteins. However, humans become ill after taking emetine for long periods because it also blocks the production of human proteins.

Tweaking emetine so that it acts only against the production of parasite proteins would make it a safer malaria treatment. To do this, scientists must first map the precise interactions between the drug and the ribosomes in parasites. Wong et al. have now used a technique called cryo-electron microscopy to examine the ribosome of the most virulent form of malaria parasite. This technique uses very cold temperatures to rapidly freeze molecules, allowing scientists to look at molecular-level details without distorting the structure of the molecule—a problem sometimes encountered in other techniques.

The images of the parasitic ribosome taken by Wong, Bai, Brown et al. show that emetine binds to the end of the ribosome where the instructions for how to assemble amino acids into a protein are copied from strands of RNA. In addition, the images revealed features of the parasitic ribosome that are not found in the human form. Drug makers could exploit these features to improve emetine so that it more specifically targets the production of proteins by the parasite and is less toxic to humans.

**DOI:** http://dx.doi.org/10.7554/eLife.03080.002

## Introduction

Malaria is responsible for an estimated 627,000 annual deaths worldwide, with the majority of victims being children under 5 years of age (WHO, 2012). At present there is no licensed malaria vaccine and parasites have developed resistance to all front-line anti-malarial drugs. As such, there is an urgent need for novel therapeutics that can be used as monotherapies or as partner drugs for combinatorial regimes (Kremsner and Krishna, 2004). An alternative to novel candidates is the repurposing or repositioning of clinically approved drugs that can be used in combination with known anti-malarials, such as chloroquine, antifolates, and artemisinin, to increase their useable lifespan by reducing resistance (Grimberg and Mehlotra, 2011).

The etiological agents for malaria are a family of unicellular protozoan pathogens of the genus *Plasmodium*. The parasite has a complex two-host lifecycle with a sexual stage occurring in the mosquito vector and an asexual stage in the human host. It is during the asexual blood stage that disease symptoms in humans first appear, including those associated with severe malaria, and it is often at this stage that the need for clinical intervention becomes apparent (Miller et al., 2002). Much of malaria pathology is the result of exponential growth of the parasite within erythrocytes, and given the critical role that protein synthesis plays in this, the translational machinery is an attractive drug target.

Protein translation in the parasite is focused on three centers (Jackson et al., 2011): the cytoplasmic ribosome, responsible for the vast majority of protein synthesis, and organellar ribosomes of the mitochondrion and non-photosynthetic plastid, termed the apicoplast (McFadden et al., 1996). In addition, and unusually for a eukaryotic cell, *Plasmodium* species have two distinct types of cytoplasmic ribosome that differ in their ribosomal RNA (rRNA) composition. These are expressed at different stages of the lifecycle, one predominantly in the mosquito vector and the other in the mammalian host, with evidence that both can occur simultaneously for limited periods (Waters et al., 1989).

Antibiotics known to target the apicoplast ribosome, such as the macrolide azithromycin, demonstrate a delayed-death effect, whereby treated parasites die in the second generation of drug exposure, and therefore have slow clinical onset (Dahl and Rosenthal, 2007 ; Goodman et al., 2007 ). However, because anti-malarial treatment at the blood-stage requires rapid intervention, we focused on the dominant, blood stage-specific cytoplasmic ribosome from the most virulent form of *Plasmodium, P. falciparum* (*Pf*80S) (Waters et al., 1989), as inhibition of cytosolic translation would be expected to be direct and fast-acting. *Pf*80S is both a candidate for development of novel therapeutics that target specific differences between itself and its counterpart in the human cytosol, and also for repurposing of anti-protozoan inhibitors, such as emetine (Matthews et al., 2013).

In this present study, we solved the structure of *Pf*80S–emetine complex at 3.2 Å resolution and built a fully-refined all-atom model. This represents, to our knowledge, the first structure of an entire eukaryotic ribosome at atomic resolution solved by electron cryo-microscopy (cryo-EM). *Pf*80S has a broad distribution of *Pf*-specific elements across its surface, with particularly long rRNA expansion segments (ESs) in the small subunit. The atomic structure of *Pf*80S in complex with emetine reveals the molecular basis of this clinically relevant anti-protozoan translation inhibitor. In doing so, we establish cryo-EM as a powerful tool for structure-based drug design.

## Results

**Figure 1.**
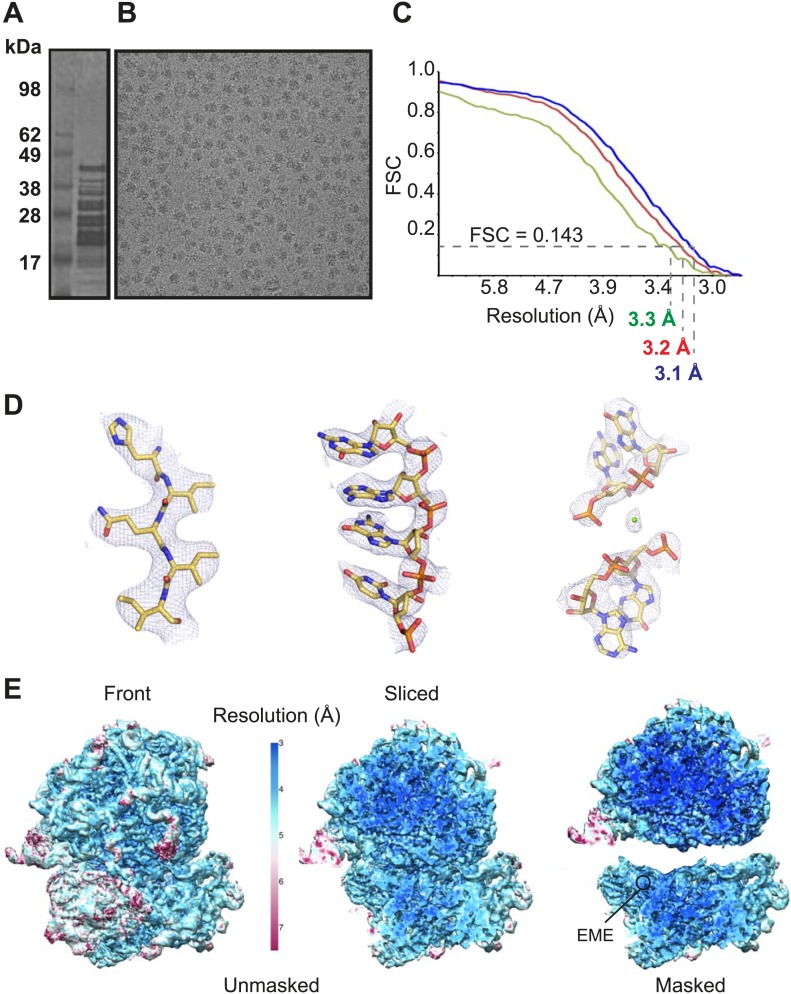
Cryo-EM data and processing. (**A**) Sucrose gradient purification of *Pf*80S ribosomes. (**B**) Representative electron micrograph showing *Pf*80S particles. (**C**) Fourier Shell Correlation (FSC) curves indicating the overall resolutions of unmasked (red), *Pf*40S masked (green) and *Pf*60S masked (blue) reconstructions of the *Pf*80S–emetine complex. (**D**) Representative density with built models of a β-strand with well-resolved side chains (left), an RNA segment with separated bases (middle), and a magnesium ion (green sphere) that is coordinated by RNA backbone phosphates. (**E**) Density maps colored according to local resolution for the unmasked *Pf*80S (left) and masked *Pf*40S and *Pf*60S subunits (right). **DOI:** http://dx.doi.org/10.7554/eLife.03080.003

Cytoplasmic ribosomes were isolated from the 3D7 strain of *P. falciparum* parasites maintained in human erythrocytes (Figure 1A,B). Limitations in parasite culture volume, yielding ∼10^10^ parasitized red blood cells and low yield of sample material (1 g of parasites yielded 0.35 mg *Pf*80S), precluded an ability to crystallize *Pf*80S to solve the structure by conventional X-ray crystallography. We therefore exploited recent advances in direct electron detection and statistical image processing (Bai et al., 2013; Allegretti et al., 2014) to determine the structure by cryo-EM at an overall resolution of 3.2 Å (Figure 1C–E, Figure 1—figure supplement 1).

**Figure 1—figure supplement 1.**
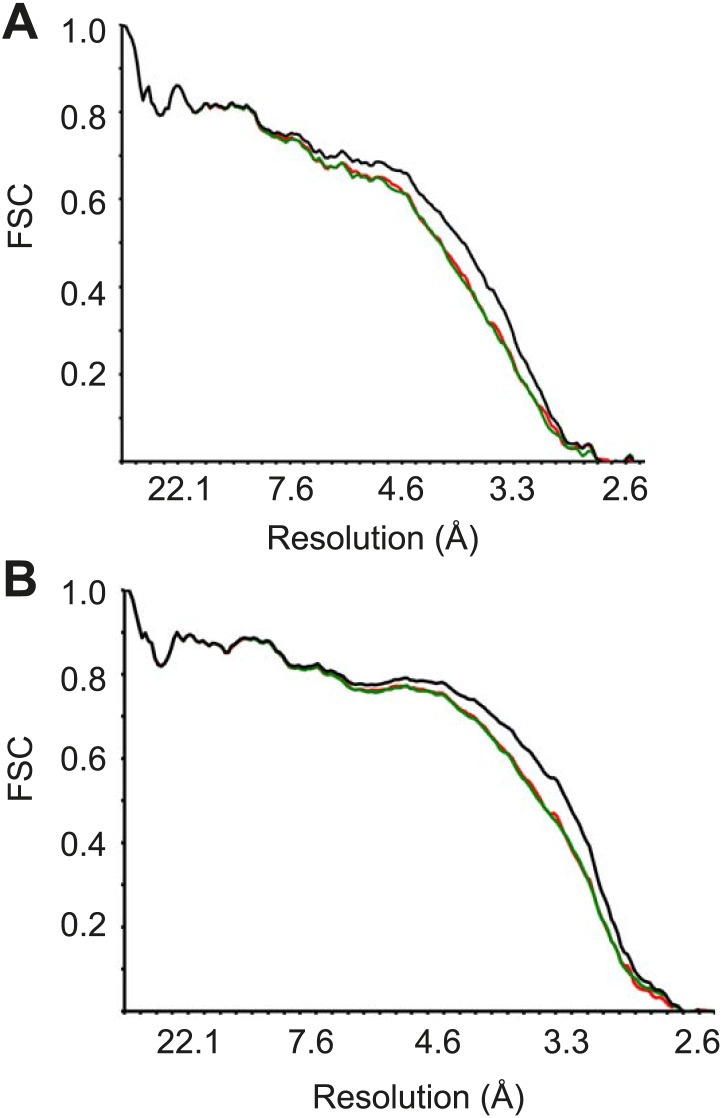
FSC curves between the final refined atomic model and the reconstructions from all particles (black); between the model refined in the reconstruction from only half of the particles and the reconstruction from that same half (FSC_work_, red); and between that same model and the reconstruction from the other half of the particles (FSC_test_, green), for *Pf*40S (A) and *Pf*60S (B). **DOI:** http://dx.doi.org/10.7554/eLife.03080.004

**Figure 2.**
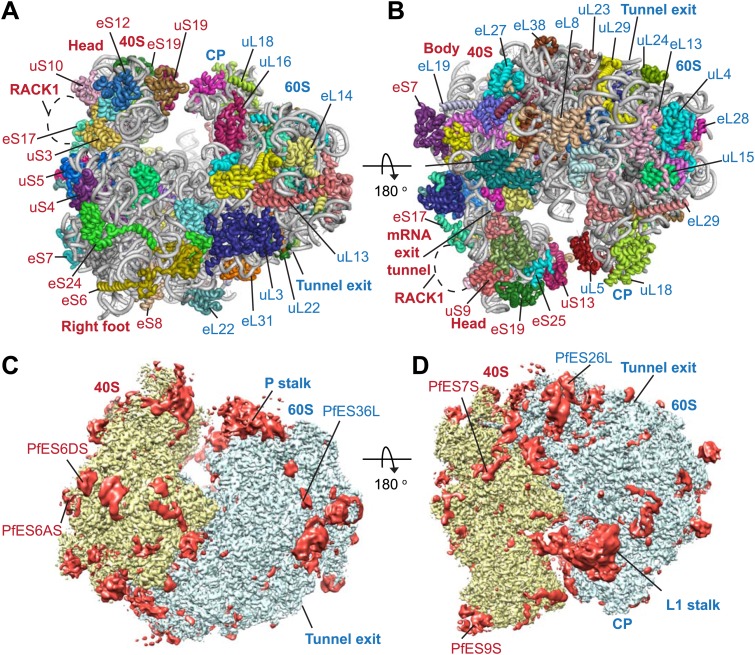
Structure of the *Pf*80S ribosome. Overview of *Pf*80S atomic model showing views facing (**A**) tRNA entry side and (**B**) tRNA exit side. rRNAs are shown in gray, proteins numbered according to Ban et al. (2014). (**C** and **D**) *Pf*40S and *Pf*60S subunits are colored in yellow and blue respectively. Flexible regions are shown in red and at a resolution of 6 Å. *Pf*-specific expansion segments (ESs) relative to human ribosomes are labeled. Their numbering is as described for the human cytoplasmic ribosome (Anger et al., 2013). **DOI:** http://dx.doi.org/10.7554/eLife.03080.005

Protein side chains and RNA bases were clearly resolved in our maps (Figure 1D). The use of model building and refinement tools that were adapted from X-ray crystallography (Amunts et al., 2014) led to a near-complete atomic model with excellent geometrical properties (Figure 2A,B; Table 1). The ribosome model comprises the large (*Pf*60S) and small subunit (*Pf*40S) with a total of 74 proteins (Tables 2 and 3) as well as the 5S, 5.8S, 18S, and 28S rRNAs and a tRNA bound at the E-site. The head region of *Pf*40S has weaker density than the rest of the ribosome due to the inherent flexibility at the neck (centered around h28). This meant that eS31, located in the beak of the 40S head (Rabl et al., 2011), could not be positioned accurately, and has therefore been omitted from the final model. Using base-pair information extracted directly from the atomic model it was possible to revise secondary structure diagrams for *P. falciparum* rRNA (Figure 2—figure supplements 1–3), facilitating comparison with rRNA of other species.

**Figure 2—figure supplement 1.**
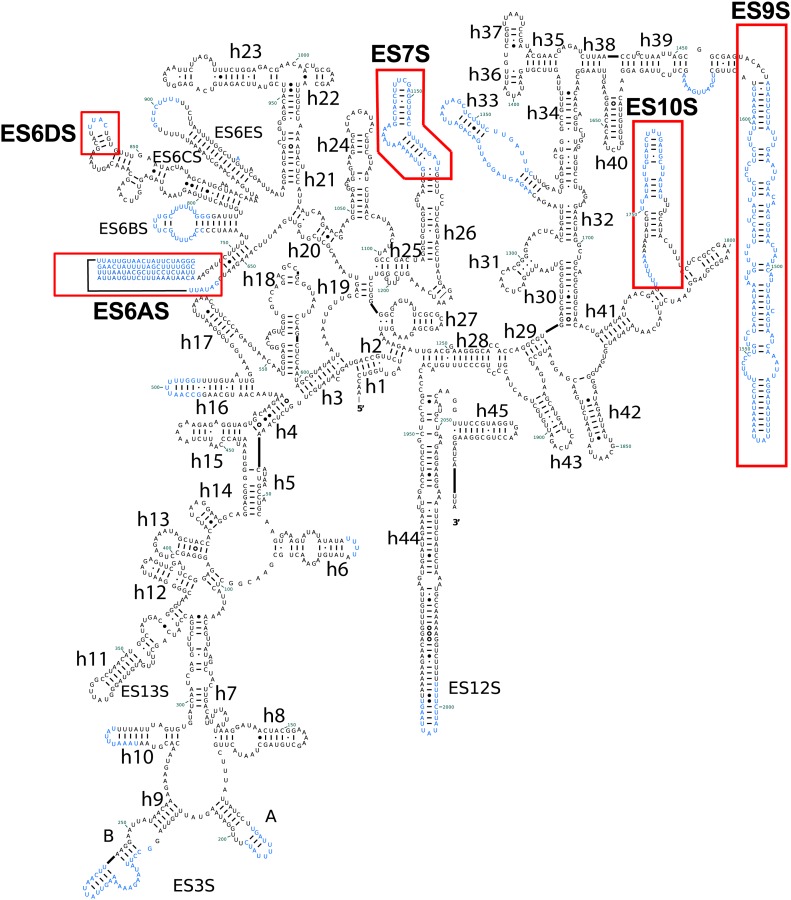
Secondary structure of *Pf*18S rRNAs. *Pf*-specific ESs are highlighted in a labeled red box. Regions not built in the atomic model are colored in blue text. The secondary structure was modified from the CRW site (Cannone et al., 2002). **DOI:** http://dx.doi.org/10.7554/eLife.03080.006

**Figure 2—figure supplement 2.**
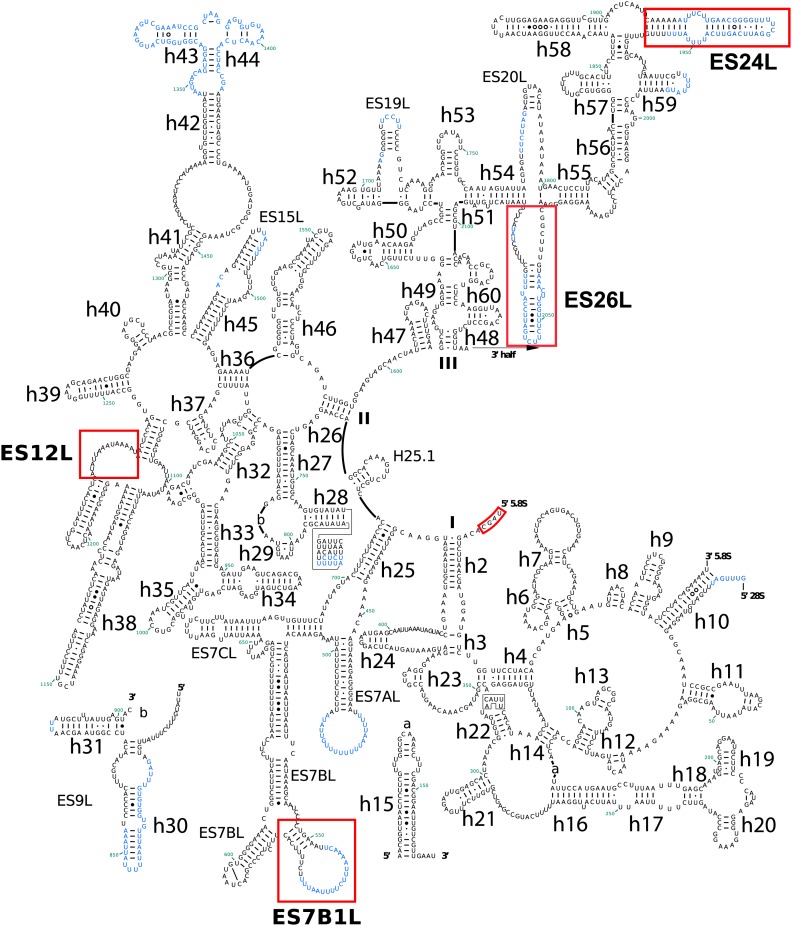
Secondary structure of the 5′ half of *Pf* 28S rRNA. *Pf*-specific ESs are highlighted in a labeled red box. Regions not built in the atomic model are colored in blue text. The secondary structure was modified from the CRW site (Cannone et al., 2002). **DOI:** http://dx.doi.org/10.7554/eLife.03080.007

**Figure 2—figure supplement 3.**
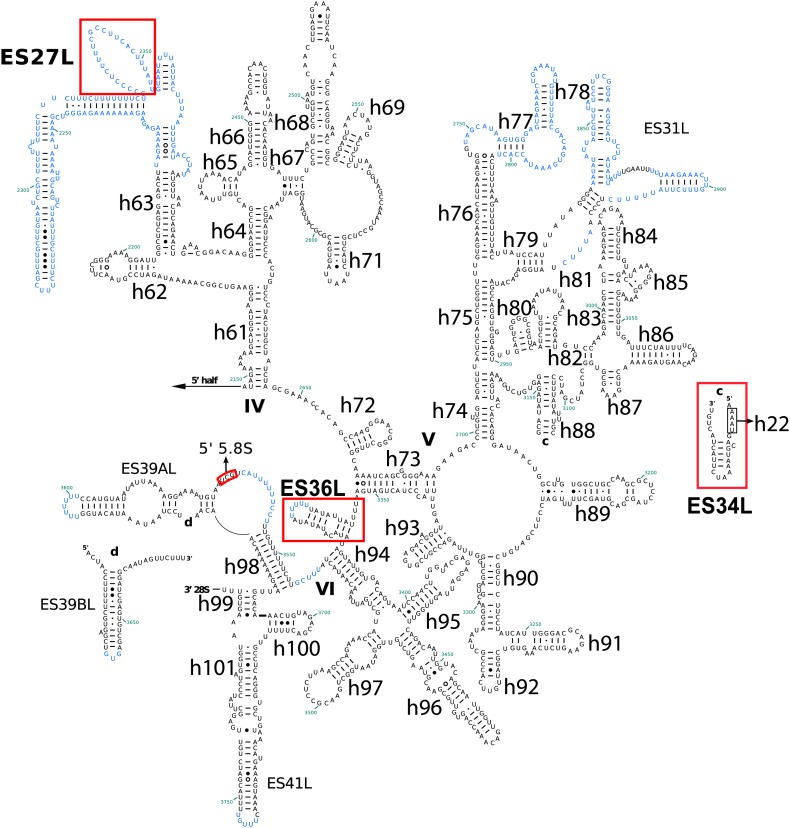
Secondary structure of the 3′ half of *Pf*28S rRNA. *Pf*-specific ESs are highlighted in a labeled red box. Regions not built in the atomic model are colored in blue text. The secondary structure was modified from the CRW site (Cannone et al., 2002). **DOI:** http://dx.doi.org/10.7554/eLife.03080.008

**Table 1.** Refinement and model statistics **DOI:** http://dx.doi.org/10.7554/eLife.03080.009

	*Pf80S–emetine*
Data collection	
Particles	105,247
Pixel size (Å)	1.34
Defocus range (μm)	0.8–3.8
Voltage (kV)	300
Electron dose (e^−^ Å^−2^)	20

	*Pf*60S	*Pf*40S
Model composition		
Non-hydrogen atoms	124,509	68,858
Protein residues	6,244	4,106
RNA bases	3,460	1,682
Ligands (Zn^2+^/Mg^2+^/emetine)	5/163/0	1/67/1
Refinement		
Resolution used for refinement (Å)	3.1	3.3
Map sharpening B-factor (Å^2^)	−60.3	−79.9
Average B factor (Å^2^)	113.1	143.2
Rfactor*	0.2294	0.257
Fourier Shell Correlation†	0.86	0.854
Rms deviations		
Bonds (Å)	0.006	0.007
Angles (°)	1.20	1.29
Validation (proteins)		
Molprobity score	2.45 (96^th^ percentile)	2.73 (95^th^ percentile)
Clashscore, all atoms	3.65 (100^th^ percentile)	4.23 (100^th^ percentile)
Good rotamers (%)	90.0	86.0
Ramachandran plot		
Favored (%)	90.4	85.4
Outliers (%)	2.4	4.2
Validation (RNA)		
Correct sugar puckers (%)	97.3	97.5
Good backbone conformations (%)	71.1	70.0

* Rfactor = Σ||F_obs_| − ||F_calc_|/Σ|F_obs_|.

† FSC_overall_ = Σ(N_shell_ FSC_shell_)/Σ(N_shell_), where FSC_shell_ is the FSC in a given shell, N_shell_ is the number of ‘structure factors’ in the shell. FSC_shell_ = Σ(F_model_ F_EM_)/(√(Σ(|F|^2^_model_)) √(Σ(F^2^_EM_)).

**Table 2.** Ribosomal proteins of the *Pf*40S subunit **DOI:** http://dx.doi.org/10.7554/eLife.03080.010

Protein names	Uniprot ID	PlasmoDB ID	Chain ID	Built residues	Extensions compared to human	Total number of residues
eS1	RS3A_PLAF7	PF3D7_0322900	B	24–233	245–262	262
uS2	RSSA_PLAF7	PF3D7_1026800	C	10–204	–	263
uS3	Q8IKH8_PLAF7	PF3D7_1465900	D	4–39; 65–78; 97–193; 207–216	–	221
uS4	Q8I3R0_PLAF7	PF3D7_0520000	E	2–186	–	189
eS4	Q8IIU8_PLAF7	PF3D7_1105400	F	2–258	–	261
uS5	Q8IL02_PLAF7	PF3D7_1447000	G	39–262	–	272
eS6	Q8IDR9_PLAF7	PF3D7_1342000	H	1–160; 170–213	249–306	306
uS7	Q8IBN5_PLAF7	PF3D7_0721600	I	7–118; 128–195	–	195
eS7	Q8IET7_PLAF7	PF3D7_1302800	J	3–190	–	194
uS8	O77395_PLAF7	PF3D7_0316800	K	2–130	–	130
eS8	Q8IM10_PLAF7	PF3D7_1408600	L	5–120; 161–213; 216–218	154–163	218
uS9	Q8IAX5_PLAF7	PF3D7_0813900	M	6–143	–	144
uS10	Q8IK02_PLAF7	PF3D7_1003500	N	21–118	–	118
eS10	Q8IBQ5_PLAF7	PF3D7_0719700	O	11–89	–	137
uS11	Q8I3U6_PLAF7	PF3D7_0516200	P	25–151	–	151
uS12	O97248_PLAF7	PF3D7_0306900	Q	2–145	–	145
eS12	RS12_PLAF7	PF3D7_0307100	R	22–78; 85–100; 111–135	10–16	141
uS13	Q8IIA2_PLAF7	PF3D7_1126200	S	12–139	–	156
uS14	C0H4K8_PLAF7	PF3D7_0705700	T	7–54	–	54
uS15	Q8IDB0_PLAF7	PF3D7_1358800	U	3–151	–	151
uS17	O77381_PLAF7	PF3D7_0317600	V	6–25; 36–161	–	161
eS17	Q8I502_PLAF7	PF3D7_1242700	W	3–83; 97–110	–	137
uS19	C0H5C2_PLAF7	PF3D7_1317800	X	21–95; 103–123	–	145
eS19	Q8IFP2_PLAF7	PF3D7_0422400	Y	15–168	1–19	170
eS21	Q8IHS5_PLAF7	PF3D7_1144000	Z	11–82	–	82
eS24	Q8I3R6_PLAF7	PF3D7_0519400	1	3–122	–	133
eS25	Q8ILN8_PLAF7	PF3D7_1421200	2	35–42; 58–84; 97–102	–	105
eS26	O96258_PLAF7	PF3D7_0217800	3	2–96	–	107
eS27	Q8IEN2_PLAF7	PF3D7_1308300	4	7–82	–	82
eS28	Q8IKL9_PLAF7	PF3D7_1461300	5	2–29; 37–66	–	67
eS30	RS30_PLAF7	PF3D7_0219200	6	6–48	–	58
eS31	Q8IM64_PLAF7	PF3D7_1402500	–	Not built	–	149

**Table 3.** Ribosomal proteins of the *Pf*60S subunit **DOI:** http://dx.doi.org/10.7554/eLife.03080.011

Protein names	Uniprot ID	PlasmoDB ID	Chain ID	Built residues	Extensions compared to human	Total number of residues
uL2	Q8I3T9_PLAF7	PF3D7_0516900	D	2–248	–	260
uL3	Q8IJC6_PLAF7	PF3D7_1027800	E	2–381	–	386
uL4	Q8I431_PLAF7	PF3D7_0507100	F	6–395	373–411	411
uL5	Q8IBQ6_PLAF7	PF3D7_0719600	G	8–51; 64–85; 92–106; 124–166	–	173
uL6	Q8IE85_PLAF7	PF3D7_1323100	H	2–186	–	190
eL6	Q8IDV1_PLAF7	PF3D7_1338200	I	9–151; 158–221	110–118; 139–143; 174–182	221
eL8	Q8ILL2_PLAF7	PF3D7_1424400	J	40–46; 54–131; 147–283	11–24;279–283	283
uL13	Q8IJZ7_PLAF7	PF3D7_1004000	K	1–201	–	202
eL13	Q8IAX6_PLAF7	PF3D7_0814000	L	2–212	134–141; 168–174	215
uL14	Q8IE09_PLAF7	PF3D7_1331800	M	8–139	–	139
eL14	Q8ILE8_PLAF7	PF3D7_1431700	N	5–150	1–18	165
uL15	C6KT23_PLAF7	PF3D7_0618300	O	2–148	–	148
eL15	C0H4A6_PLAF7	PF3D7_0415900	P	2–205	–	205
uL16	Q8ILV2_PLAF7	PF3D7_1414300	Q	2–101; 118–206	–	219
uL18	Q8ILL3_PLAF7	PF3D7_1424100	R	5–126; 141–185; 189–250; 271–293	–	294
eL18	C0H5G3_PLAF7	PF3D7_1341200	U	5–184	–	184
eL19	C6KSY6_PLAF7	PF3D7_0614500	T	2–182	–	182
eL20	Q8IDS6_PLAF7	PF3D7_1341200	S	2–187	–	184
eL21	Q8ILK3_PLAF7	PF3D7_1426000	V	4–158	–	161
uL22	Q8IDI5_PLAF7	PF3D7_1351400	W	4–154; 197–215	–	203
eL22	Q8IB51_PLAF7	PF3D7_0821700	X	40–136	4–18; 34–38	139
uL23	Q8IE82_PLAF7	PF3D7_1323400	Y	88–188	13–34; 57–67	190
uL24	O77364_PLAF7	PF3D7_0312800	Z	2–122	–	126
eL24	Q8IEM3_PLAF7	PF3D7_1309100	0	8–69	–	162
eL27	Q8IKM5_PLAF7	PF3D7_1460700	1	2–126;132–146	–	146
eL28	Q8IHU0_PLAF7	PF3D7_1142500	2	2–69; 77–82; 86–98; 103–119	–	127
uL29	Q8IIB4_PLAF7	PF3D7_1124900	3	3–121	–	124
eL29	C6S3J6_PLAF7	PF3D7_1460300	4	2–67	–	67
uL30	O97250_PLAF7	PF3D7_0307200	5	35–257	–	257
eL30	Q8IJK8_PLAF7	PF3D7_1019400	6	8–105	–	108
eL31	Q8I463_PLAF7	PF3D7_0503800	7	15–88; 95–116	–	120
eL32	Q8I3B0_PLAF7	PF3D7_0903900	8	2–126	–	131
eL33	Q8IHT9_PLAF7	PF3D7_1142600	9	35–137	1–35	140
eL34	Q8IBY4_PLAF7	PF3D7_0710600	a	2–107	–	150
eL36	Q8I713_PLAF7	PF3D7_1109900	b	2–27; 38–106	5–10	112
eL37	C0H4L5_PLAF7	PF3D7_0706400	c	2–90	–	92
eL38	Q8II62_PLAF7	PF3D7_1130100	d	2–31; 36–77	–	87
eL39	C0H4H3_PLAF7	PF3D7_0611700	e	2–30; 38–51	–	51
eL40	Q8ID50_PLAF7	PF3D7_1365900	f	1–51	–	52
eL41	C6S3G4_PLAF7	PF3D7_1144300	g	3–39	1–14	39
eL43	RL37A_PLAF7	PF3D7_0210100.1	h	2–86	–	96
eL44	RL44_PLAF7	PF3D7_0304400	i	2–96	–	104

Currently, high resolution structures of eukaryotic ribosomes have been solved using X-ray crystallography and are limited to just three structures; the individual subunits from a ciliated protozoan, *Tetrahymena thermophila* (Klinge et al., 2011; Rabl et al., 2011), and the complete 80S ribosome from the yeast *Saccharomyces cerevisiae* (Ben-Shem et al., 2011). These models have been used to interpret lower resolution structures solved by cryo-EM of other species including the yeast *Kluyveromyces lactis* (Fernandez et al., 2014), *Drosophila melanogaster* (Anger et al., 2013), *Trypanosoma brucei* (Hashem et al., 2013), as well as human ribosomes (Anger et al., 2013) and provide the basis of the nomenclature used for describing the structures.

To examine overall architectural differences, we compared the model of *Pf*80S to yeast 80S (Ben-Shem et al., 2011). Perhaps the largest difference is the absence of RACK1 (Figure 1A,B), which associates with the head of the 40S in the vicinity of the mRNA exit channel (Sengupta et al., 2004; Rabl et al., 2011) and has been identified in all eukaryotic ribosome structures solved to-date. RACK1 serves as a signaling scaffold that can recruit other proteins to the ribosome and may link the ribosome with signal transduction pathways, thus allowing translation regulation in response to stimuli. It has also been proposed that RACK1 promotes the docking of ribosomes at sites where local translation is required (Nilsson et al., 2004).

*Pf*RACK1 is conserved with its human homolog with an identity of 60%. The binding site on the ribosome, which comprises eS17, uS3, and 18S rRNA helices h39 and h40 (Figure 1A,B), also appears highly conserved (Rabl et al., 2011). However, the C-terminus of uS3 is not resolved in our structure and probably only becomes ordered upon binding RACK1. The absence of *Pf*RACK1 as an integral member of the small subunit indicates either a different mode of interaction between the ribosome and *Pf*RACK1 in *Plasmodium* compared to humans, or that under the culturing conditions used *Pf*RACK1 is not expressed, or expressed in a form that does not interact with the ribosome. In yeast, RACK1 has been shown to be present in both a ribosome- and a non-ribosome-bound form dependent on growth conditions (Baum et al., 2004). If the interaction between *Pf*RACK1 and the *Pf*40S is weaker than in other organisms, the possibility that *Pf*RACK1 dissociated during purification and grid preparation cannot be discounted.

The yeast 80S structure was also solved in the presence of STM1, a translation repressor protein, that binds to the head region of the 40S and blocks mRNA entry and binding of tRNA to the A- and P-sites (Rabl et al., 2011). The human and *D. melanogaster* structures also co-purified with an STM1-like protein (SERBP1 and VIG2 respectively) (Anger et al., 2013). *Pf*80S is not bound by a suppressor molecule, as also observed for the *T. brucei* structure (Hashem et al., 2013), and hence reflects a ribosome capable of active translation.

*Pf*80S co-purifies with a tRNA bound to the E-site. Although the density is not well resolved, presumably as a result of low and mixed occupancy, it could be interpreted by positioning a model of tRNA^Met^. The presence of tRNA helps to partially stabilise the L1 stalk near the elbow of the tRNA, however the stalk remains considerably flexible and is averaged out of the high-resolution reconstruction.

Perhaps due to the absence of RACK1 and/or STM1 or the presence of an E-site tRNA, the head of *Pf*40S adopts an orientation with respect to the body that is different to the yeast structure, with uS11 at the beak of the small subunit displaced by more than 10 Å. The root mean square deviation (RMSD) of the two small subunits is 2.9 Å^2^, however if the head and body are superimposed independently this improves to 1.0 Å^2^ and 1.5 Å^2^ respectively. The structure of *Pf*60S superimposes with the yeast 60S with a RMSD of 1.6 Å^2^. The largest morphological differences in this subunit result from a cluster of rRNA helices (ES7AL, ES15L, and ES7CL) protruding at the solvent side.

Given the potential of *Pf*80S as a drug target, we sought to describe its detailed structure in comparison to its direct counterpart in the human cytoplasm, where a 4.8 Å cryo-EM 80S structure represents the highest resolution solved to-date (Anger et al., 2013). Therefore, all protein extensions and rRNA expansion segments (ESs) are annotated on the basis of comparison with human ribosomes. While the core of the *Pf*80S and human ribosome are conserved, the periphery of the ribosomes differs extensively in the nature and length of rRNA ESs and protein extensions. The constraints on rRNA expansion appear to be fewer than on protein extension, as rRNA contributes greater to the mass difference between species.

Compared to human ribosomes, *P. falciparum* typically has shorter ESs, some of which are entirely absent in the large subunit (ES7D-HL, ES9AL, ES10L, ES20L, ES30L) (Table 4). The functions, if any, of many of these ESs are not well known. ES7E, which is highly conserved in vertebrates, is implicated in selenoprotein synthesis by binding the SBP2 protein that specifically recruits the selenocysteine-specific tRNA and elongation factor (Kossinova et al., 2014). While *P. falciparum* does utilize selenocysteine, it is incorporated into very few proteins (Lobanov et al., 2006) and there is no homolog of SBP2, providing a possible explanation for why ES7E is not present in *Plasmodium*.

**Table 4.** Comparison of ESs in *Pf*80S and human cytoplasmic ribosomes **DOI:** http://dx.doi.org/10.7554/eLife.03080.012

rRNA	ES	Helix	Comparison between *Pf*80S and human ribosomes
18S	ES2S		Shorter loop in *Pf*80S
	ES3S	A	Conserved
		B	Truncated in *Pf*80S
	ES13S		Conserved
	ES6S	A	Expanded in *Pf*80S
		B	Truncated in *Pf*80S
		C	Conserved
		D	Expanded in *Pf*80S
		E	Conserved
	ES7S		Expanded in *Pf*80S
	ES14S		Conserved
	ES9S		Expanded in *Pf*80S
	ES10S		Expanded in *Pf*80S
	ES12S		Helix truncated in *Pf*80S
28S	ES3L		Conserved
	ES4L		Conserved
	ES5L		Conserved
	ES7L	A	Truncated in *Pf*80S
		B	Truncated. Loop in *Pf*80S forms a novel interaction with eL14
		B1	*Pf*-specific ES
		C	Present
		D–H	Absent from *Pf*80S
	ES8L	H28	Expanded in *Pf*80S
	ES9L	A	Absent in *Pf*80S
		H30	Conserved
		H31	Conserved
	ES10L		Absent in *Pf*80S
	ES12L		Expanded in *Pf*80S
	ES15L	A	Truncated in *Pf*80S
	ES19L		Truncated in *Pf*80S
	ES20L	A	Absent in *Pf*80S
		B	Conserved in *Pf*80S
	ES26L		Expanded in *Pf*80S
	ES27L	A–C	Not present in Pf80S model, predicted divergence between *Pf* and human cytoplasmic ribosomes
	ES30L		Absent in *Pf*80S
	ES31L	A	Conserved
		B	Expanded in *Pf*80S
		C	Conserved
	ES34L		*Pf*-specific ES
	ES36L		*Pf*-specific ES
	ES39L	A	Conserved; preceding loop in *Pf*80S forms a short helix (3 base pairs) with the 5′ end of the 5.8S rRNA
		B	Conserved
	ES41L		Conserved

The largest *Pf-*specific ESs are concentrated in the 18S rRNA, with ES6S and ES9S being particularly extended (Figure 2C,D; Figure 2—figure supplement 1). These ESs, like those described in both the human (Anger et al., 2013) and *Trypanosoma brucei* (Hashem et al., 2013) ribosome structures, are highly flexible and, in our structure, are only partly visible using a map filtered at 6 Å (Figure 2C,D). We have therefore not included these sections in our atomic model. ES10S is located at the top of the 40S head and has been partially built.

*P. falciparum* ribosomes resemble those of *T. brucei* in that both have large ES6S and ES7S, although these are slightly larger in *T. brucei* (Hashem et al., 2013). ES6S is in contact with ribosomal components that form part of the mRNA entry and exit sites and was therefore suggested as being involved in translation initiation (Jenner et al., 2012). Recently, ES6/7S have been implicated in binding of the conserved translation initiation factor eIF3 based on superposition with a mammalian 43S complex (Hashem et al., 2013). Almost 90 nucleotides of ES6AS are averaged out of our high-resolution reconstruction indicating this stalk is highly flexible, perhaps acting in a manner similar to the P stalk (known as the L7/L12 stalk in prokaryotes) by recruiting factors necessary for translation (in this case eIF3). The other large ES of the 18S rRNA, ES9S, is positioned at the head of the 40S. Given both the intrinsic mobility of the head and presumably the ES itself, there is no density for this ∼150 nucleotide *Pf*-specific element and the role it plays remains unclear.

**Figure 3.**
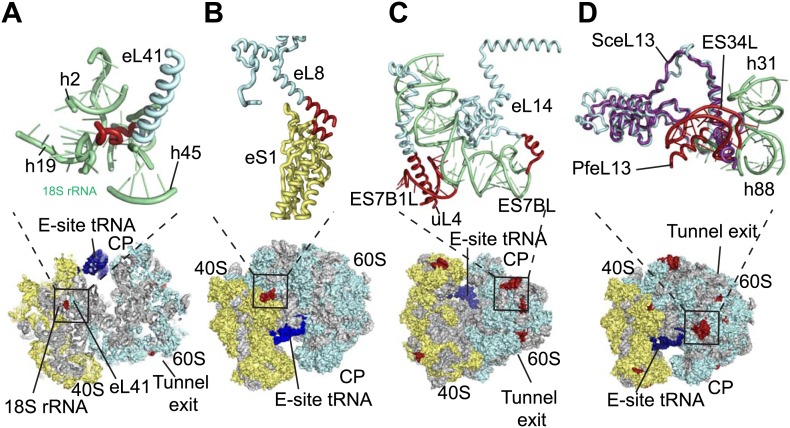
Details of *Pf*-specific protein extensions and rRNA ESs near the (A and B) subunit interface (C) P stalk and (D) the L1 stalk. *Pf*-specific elements are shown in red. **DOI:** http://dx.doi.org/10.7554/eLife.03080.013

The sites of *Pf-*specific elements are broadly distributed across the solvent-accessible surface of the ribosome, although the region surrounding the exit tunnel is conserved (Klinge et al., 2011) and undecorated with ESs and protein extensions (Figure 2C,D). The subunit interface and eukaryotic-specific bridges, which in addition to having structural roles help transmit information to coordinate activity during translation (Ben-Shem et al., 2011), are generally highly conserved in *Pf*80S. There are a couple of examples of stabilizing interactions that are not observed in human ribosomes. Firstly, eL41, the smallest ribosomal protein, bridges the two subunits (Ben-Shem et al., 2011) and has a 14-residue *Pf*-specific N-terminal extension that reaches into a pocket formed by 18S rRNA of the small subunit and tightly anchors the protein (Figure 3A). Secondly, an additional small bridge (∼200 Å^2^) is formed between the platform of *Pf*40S and the region around the L1 stalk by the C-terminal helix extension of eL8 interacting with the C-terminal helix of eS1 (Figure 3B).

Further ordered *Pf-*specific elements are concentrated near the L1 and P stalks of *Pf*60S. Directly above the P stalk, the *Pf-*specific ES7B1L forms a diverted part of ES7CL that is stabilized by several electrostatic interactions with a C-terminal helix extension of uL4 (Figure 3C). Towards the back of the P-stalk, the C-terminal helix extension of eL14 caps the stem loop of ES7BL (Figure 3C). On the opposite side of the ribosome, near the E-site tRNA, the *Pf-*specific stem loop ES34L is positioned directly above the L1 stalk (Figure 3D). This ES appears to have caused a 60° rotation of the C-terminal helix of eL13 relative to its position in human ribosomes (Figure 3D). The tip of the helix is displaced by ∼28 Å away from the L1 stalk and now stabilizes the interaction between ES34L and the loop of h22. Since the L1 stalk is required for coordinating the movement of tRNAs and the P stalk is required for coordinating the movement of translation factors during the various steps of protein synthesis (Gonzalo and Reboud, 2003), the expanded mass around the stalks of *Pf*80S may have functional implications for translation in *P. falciparum*.

**Figure 4.**
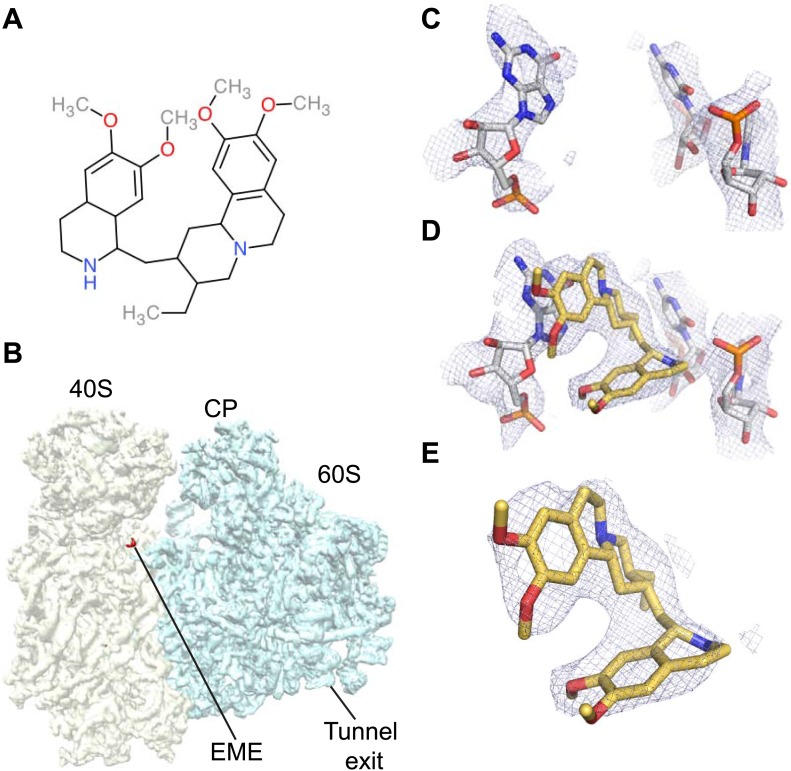
Emetine binds to the E-site of the *Pf*40S subunit. (**A**) 2D chemical structure of emetine. (**B**) A 4.5 Å filtered difference map (red density) at 5 standard deviation overlaid with the *Pf*80S map filtered at 6 Å (blue and yellow for *Pf*60S and *Pf*40S respectively), showing the emetine density at the E-site of the *Pf*40S. The emetine binding site in (**C**) empty and (**D**) emetine-bound structures, with (**E**) density for emetine alone at 3.2 Å. **DOI:** http://dx.doi.org/10.7554/eLife.03080.014

The ability to determine atomic-resolution structures of *Pf*80S provides a platform for investigating the action of anti-malarial therapeutics that target the ribosome. The clinically used, broad-spectrum eukaryotic translation inhibitor emetine (Figure 4A) (Grollman, 1968), has been reported to act as a translocation inhibitor targeting the ribosome (Jimenez et al., 1977; Dinos et al., 2004), although its precise mode of action is unknown. Emetine is a natural product alkaloid from the plant *Carapichea ipecacuanha*, and an approved medicine for the treatment of amoebiasis (Goodwin et al., 1948). Although its toxicity associated with chronic usage in humans has limited its clinical use against malaria in its current formulation (Dempsey and Salem, 1966), emetine does demonstrate potent antimalarial activity with a 50% inhibitory concentration (IC_50_) of 47 nM against the blood stage of multidrug resistant strains of *P. falciparum* (Matthews et al., 2013). Moreover, the immediate therapeutic effect it offers by rapid killing of blood stage parasites may warrant re-consideration of the use of emetine or its derivatives for short periods during acute malaria infection (James, 1985).

**Figure 5.**
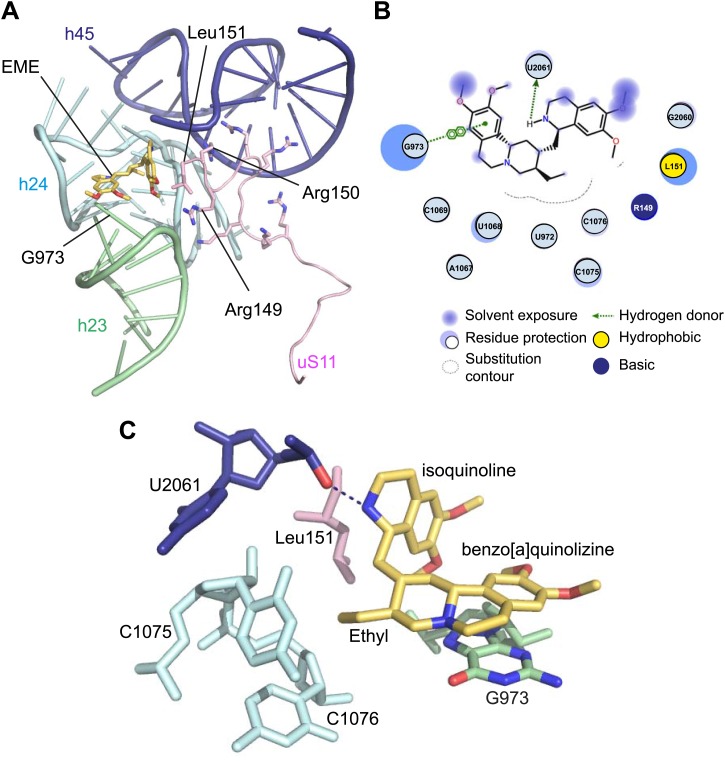
Molecular details of the emetine–ribosome interaction. (**A**) Overview of emetine at the binding interface formed by the three conserved rRNA helices and uS11. h23 is in green, h24 in cyan, h45 in blue, uS11 in pink, and emetine in yellow. (**B**) 2D representation showing the interaction of emetine with binding residues. Substitution contour represents potential space for chemical modification of emetine. (**C**) Residues in physical contact with emetine. Hydrogen bond is indicated as dashes. **DOI:** http://dx.doi.org/10.7554/eLife.03080.015

Incubation of purified *Pf*80S with a 1 mM emetine solution prior to cryo-EM grid preparation, led to a 3.2 Å resolution structure of the complex. Using soft masking, the resolution for the large subunit improved to 3.1 Å, with the small subunit at 3.3 Å (Figure 1C). A difference map was calculated from the reconstructions with and without emetine and showed a single, continuous feature near the E-site of *Pf*40S with a shape and size congruent with a single emetine molecule when thresholded at 5 standard deviations, and with a maximum value of 11 standard deviations (Figure 4B). At this position in our map, the density provided sufficient detail to confidently model the emetine molecule (Figure 4C–E). The emetine binding pocket is formed at the interface between 18S rRNA helices 23, 24, 45, and the C-terminus of uS11 (Figure 5A). Comparison with the unliganded map showed that binding of emetine does not induce changes to the pocket (Figure 4C,D). The benzo[a]quinolizine ring of emetine mimics a base-stacking interaction with G973 of h23 and its ethyl group forms a hydrophobic interaction with C1075 and C1076 of h24, whereas the isoquinoline ring is stacked against the C-terminal Leu151 of uS11 (Figure 5B,C). The interaction is stabilized by a hydrogen bond formed between the NH group of the isoquinoline ring in emetine and an oxygen atom on the backbone of U2061 of h45 (Figure 5B,C). Although there is no high-resolution structure of the human cytoplasmic ribosome, comparison of the emetine binding site in *Pf*80S with the equivalent region in the 4.8 Å human structure (Anger et al., 2013) revealed that each of the core binding elements are conserved (Figure 5—figure supplement 1) indicating that emetine likely binds to the cytoplasmic host ribosomes in the same way, potentially accounting for the observed cytotoxicity in humans.

**Figure 5—figure supplement 1.**
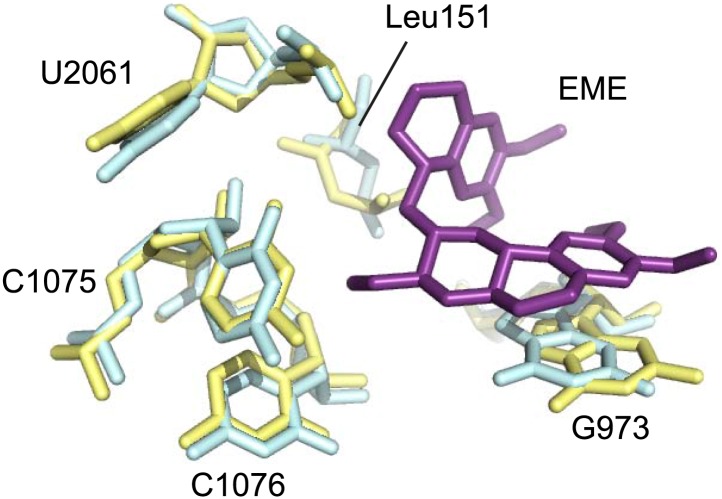
Comparison of the emetine binding residues between *Pf*80S and human ribosomes. Human and *Pf*-specific elements are colored in yellow and cyan respectively, with *Pf* numbering. Emetine is in purple. **DOI:** http://dx.doi.org/10.7554/eLife.03080.016

The identified binding site is consistent with mutations of Arg149 and Arg150 of uS11 in Chinese hamster ovary (CHO) cells that have been found to confer resistance to emetine (Madjar et al., 1982). At the emetine-binding pocket, h24 is sandwiched between the apexes of h23 and h45. The C-terminus of uS11 adopts a long coil with seven basic residues (residues 141–151; RKKSGRRGRRL), which form electrostatic interactions with the phosphate backbones of h45, h23 and h24, thereby stabilizing the conformation of this coil together with the 18S rRNA (Figure 5A). This would explain the molecular basis for resistance whereby mutations of the C-terminal arginine residues of uS11 destabilize h23 and h45, disrupting the binding pocket.

**Figure 6.**
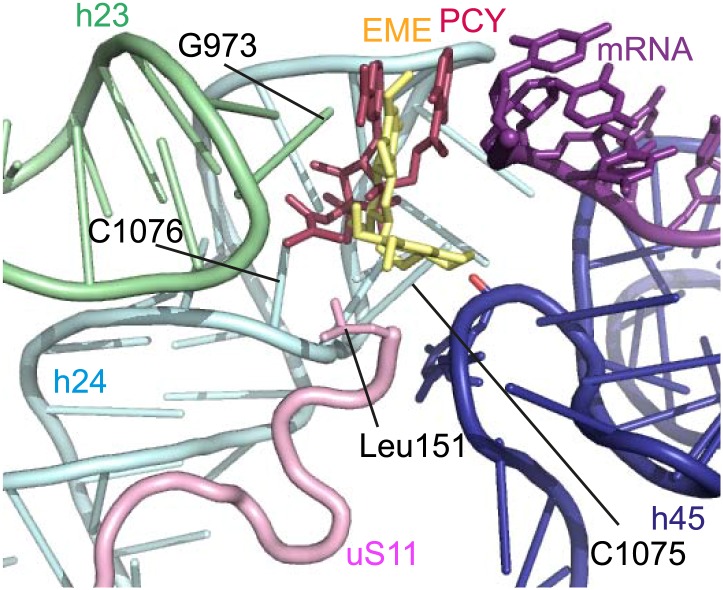
Comparison with pactamycin. Superposition of emetine and pactamycin at the *Pf*40S emetine binding pocket. Emetine and pactamycin are shown in yellow and red respectively. **DOI:** http://dx.doi.org/10.7554/eLife.03080.017

The mode of binding of emetine resembles the way in which pactamycin, previously thought to be a unique class of antibiotic, binds to the bacterial 30S (Brodersen et al., 2000). In both structures the guanine base at the tip of h23 (G973 in *Pf*; G693 in bacteria) forms a stacking interaction with the hydrophobic rings of either compound. Moreover, the two cytosine bases of h24 (C1075 and 1076 in *Pf;* C795 and 796 in bacteria) are each involved in drug binding (Brodersen et al., 2000; Figure 6). The hydrogen bond to the backbone of h45 and the hydrophobic interaction with Leu151 of uS11 are specific to the *Pf*80S–emetine interaction. In the 30S-pactamycin complex, the last base of the E-site codon of the mRNA was displaced 12.5 Å compared to the native path of mRNA (Brodersen et al., 2000) thereby blocking mRNA/tRNA entry into the E-site during the translocation step of protein synthesis (Dinos et al., 2004). Based on these structures, emetine appears to elicit its inhibitory effect by the same mechanism as pactamycin.

## Discussion

The resolution revolution in cryo-EM (Kühlbrandt, 2014) is the product of a new generation of sensors that detect electrons directly (without first converting to light) and have improved quantum efficiencies. These cameras are fast enough to follow beam-induced movement of the particles caused by irradiation with electrons. Statistical movie processing can compensate for this movement allowing for structures to be solved at atomic precision. We have harnessed these technological advances to determine the first structure of a ribosome from a parasite at atomic resolution. Previously, structures of eukaryotic cytosolic 80S ribosomes at a similar resolution had only been possible using X-ray crystallography (Ben-Shem et al., 2011). From the reconstruction of *Pf*80S–emetine complex at 3.2 Å, we determined a stereochemically accurate all-atom model using recent developments in model building, refinement, and validation (Amunts et al., 2014).

The structure of *Pf*80S further demonstrates the diversity of ribosome structures among eukaryotes, especially in terms of the location and nature of ESs at the periphery, while maintaining a conserved core. The observation of *Pf*-specific features could serve as the basis for exploring their functional relevance as an essential, first step towards finding efficacious and clinically safe anti-malarial drugs. An alternative to drug development against *Pf-*specific ribosomal elements is the repurposing of existing antibiotics as anti-malarials. By determining the structure of *Pf*80S in both a liganded and unliganded state, we were able to locate the binding site of the anti-protozoan inhibitor, emetine, using an unbiased difference map. That emetine and pactamycin share a binding pocket in eukaryotic ribosomes could not be predicted based on the chemical structures of the drug molecules only. Pactamycin itself has been shown to have potent antiprotozoal activity against both drug-susceptible and drug-resistant strains of *P. falciparum* (Otoguro et al., 2010). Chemical modifications to pactamycin have yielded analogs that maintain antimalarial activity but with reduced cytotoxicity against mammalian cells (Lu et al., 2011). Similarly, an emetine derivative, dehydroemetine, which differs by the presence of a double bond next to the ethyl group of benzo[a]quinolizine ring, exhibits less toxic effects than the parental compound while maintaining anti-parasitic properties (Dempsey and Salem, 1966; Chintana et al., 1986). This suggests that compounds targeting the emetine/pactamycin binding site are amenable to optimization, potentially leading to drugs more suited to clinical use. The *Pf*80S–emetine structure reveals an edge centered on the ethyl group of the molecule that could be subjected to modification to increase the affinity of emetine for the binding pocket (Figure 5B, labelled as the ‘contour edge’). Although based on the similarity with the binding site in humans it is unlikely that emetine can be structurally modified to not bind the mammalian system, as demonstrated in the case of dehydroemetine modifications can reduce its cytotoxicity. Although the mechanism for such reduced cytotoxicity mediated by pactamycin and emetine analogs is not known, it may be possible that these derived compounds selectively target tumor/parasite cells that are rapidly dividing, whereby protein synthesis is more sensitive to drug action in these cells. As in the case of antibiotics repurposed as antitumor agents, there is a clinical role for eukaryotic antibiotics that target systems with differential rates of translation provided usage is carefully directed. In malaria, eukaryotic antibiotics, such as emetine, could be used in combination with the slow-acting, but more specific apicoplast-targeting antibiotics (Dahl and Rosenthal, 2007).

This work demonstrates the power of contemporary cryo-EM for drug discovery. A drug, with a previously unknown binding site, can be visualized inside a macromolecular complex that is almost 10,000 times larger in molecular weight and at a level of detail comparable to that obtained by X-ray crystallography. By avoiding the need for crystallization one of the bottlenecks of solving a structure is bypassed. It allows structures to be solved from very small sample quantities, with sample heterogeneity improved through image processing. As such, cryo-EM is of particular use for solving the structures of macromolecules in their native state, isolated from pathogenic organisms where culturing large quantities is not possible.

In summary, our cryo-EM analyses reveal the first structure of a ribosome from a parasite at atomic resolution, along with detailed insights into the molecular basis of a known anti-protozoan translation inhibitor. Finally, it demonstrates that cryo-EM offers an attractive route towards the development of new compounds that target macromolecules by facilitating structure–activity relationships in otherwise intractable biological systems.

## Materials and methods

### Parasite culture and ribosome purification

Wild-type 3D7 strain of *P. falciparum* parasites were maintained in human erythrocytes (blood group O) at a hematocrit of 4% with 10% Albumax. Saponin lysed parasite pellets were incubated with lysis buffer (20 mM Hepes, pH 7.4, 250 mM KCl, 25 mM Mg(CH_3_COO)_2_, 0.15% Triton, 5 mM 2-mecaptoethanol) at 4°C for 1 hr. Ribosomes were purified by ultracentrifugation initially with a sucrose cushion (20 mM Hepes pH 7.4, 1.1 M sucrose, 40 mM KCH_3_COO, 10 mM NH_4_CH_3_COO, 10 mM Mg(CH_3_COO)_2_, and 5 mM 2-mecaptoethanol) followed by a 10–40% sucrose gradient separation step using the same buffer.

### Electron microscopy

Aliquots of 3 μl of purified *Pf*80S at a concentration of ∼160 nM (∼0.5 mg/ml) were incubated for 30 s on glow-discharged holey carbon grids (Quantifoil R1.2/1.3), on which a home-made continuous carbon film (estimated to be ∼30 Å thick) had previously been deposited. Grids were blotted for 2.5 s and flash frozen in liquid ethane using an FEI Vitrobot. For the empty *Pf*80S sample, grids were transferred to an FEI Titan Krios electron microscope that was operated at 300 kV. Images were recorded manually during two non-consecutive days on a back-thinned FEI Falcon II detector at a calibrated magnification of 135,922 (yielding a pixel size of 1.03 Å). Defocus values in the final data set ranged from 0.7 to 3.9 µm.

To prepare the *Pf*80S–emetine sample, purified *Pf*80S at 160 nM was incubated with a 1 mM solution of emetine in 20 mM Hepes pH7.4, 40 mM KCH_3_COO, 10 mM NH_4_CH_3_COO, 10 mM Mg(CH_3_COO)_2_, and 5 mM 2-mecaptoethanol for 15 min at 25°C prior to blotting and freezing as described above. *Pf*80S–emetine grids were transferred to an FEI Tecnai Polara electron microscope that was operated at 300 kV. Images were recorded manually during two non-consecutive days on a back-thinned FEI Falcon II detector at a calibrated magnification of 104,478 (yielding a pixel size of 1.34 Å). Defocus values in the final data set ranged from 0.8 to 3.8 µm.

During the data collection sessions of both samples, all images that showed signs of significant astigmatism or drift were discarded. An in-house built system was used to intercept the videos frames from the detector at a rate of 17 s^−1^ for the Krios and 16 s^−1^ for the Polara microscope.

### Image processing

We used RELION (version 1.3-beta) for automated selection of 126,727 particles from 1310 micrographs for the empty *Pf*80S sample; and 158,212 particles from 1081 micrographs for the *Pf*80S–emetine sample. Contrast transfer function parameters were estimated using CTFFIND3 (Mindell and Grigorieff, 2003). All 2D and 3D classifications and refinements were performed using RELION (Scheres, 2012). To discard bad particles, we used a single round of reference-free 2D class averaging with 100 classes for both data sets, and a single round of 3D classification with four classes for the *Pf*80S–emetine data set. The final refinement for the empty *Pf*80S and *Pf*80S–emetine sample contained 72,293 and 105,247 particles, respectively. A 60 Å low-pass filtered cryo-EM reconstruction of the yeast cytoplasmic 80S ribosome (EMDB-2275 [Ben-Shem et al., 2010]) was used as an initial model for the 3D refinement.

For the correction of beam-induced movements, we used statistical movie processing as described previously (Bai et al., 2013), with running averages of five movie frames, and a standard deviation of 1 pixel for the translational alignment. To further increase the accuracy of the movement correction, we used the beta version of RELION-1.3 to fit linear tracks through the optimal translations for all running averages, and included neighboring particles on the micrograph in these fits. In addition, we employed a resolution and dose-dependent model for the radiation damage, where each frame is weighted with a different B-factor as was estimated from single-frame reconstructions. These procedures yielded maps with an overall resolution of 3.4 Å for the empty *Pf*80S and 3.2 Å for *Pf*80S–emetine.

Reported resolutions are based on the gold-standard FSC = 0.143 criterion (Chen et al., 2013) and were corrected for the effects of a soft mask on the FSC curve using high-resolution noise substitution (Chen et al., 2013). Soft masks were made by converting atomic models into density maps, binarizing those, and adding cosine-shaped edges. Prior to visualization, all density maps were corrected for the modulation transfer function (MTF) of the detector, and then sharpened by applying a negative B-factor (Table 1) that was estimated using automated procedures (Rosenthal and Henderson, 2003).

In order to locate emetine in the *Pf*80S–emetine reconstruction, we calculated a difference map between the reconstructions of empty *Pf*80S and *Pf*80S–emetine. To this purpose, the two MTF-corrected and B-factor sharpened maps were aligned with respect to each other using the ‘Fit in Map’ functionality in UCSF Chimera (Pettersen et al., 2004 ), and the empty *Pf*80S map was re-interpolated on the Cartesian grid of the *Pf80S–emetine* map prior to subtraction of the maps in RELION. For visualization purposes, the resulting difference map was low-pass filtered at 4.5 Å and the threshold was set at 5 standard deviations as calculated within the area of the *Pf*80S ribosome (Figure 4B). At this threshold, only one continuous U-shaped feature was visible. The highest difference density inside this feature extended to 11 standard deviations in the difference map.

Local resolution variations in all reconstructions were estimated using ResMap (Kucukelbir et al., 2014). Presumably due to unresolved structural heterogeneity the local resolution in the small ribosomal subunit was typically worse than in the large ribosomal subunit. Therefore, for the *Pf80S–emetine* structure, we performed two additional ‘focussed’ refinements, where we masked out the large or the small subunit at every iteration. This gave rise to two maps (Figure 1E) with improved density for either the small subunit (at an overall resolution of 3.3 Å) or the large ribosomal subunit (at an overall resolution of 3.1 Å), and these maps were used for the refinement of the atomic model as described below.

### Model building and refinement

Ribosomal protein sequences from the 3D7 strain of *P. falciparum* were taken from *PlasmoDB* (The Plasmodium Genome Database Collaborative, 2001) and used as template sequences to obtain homology models generated from I-TASSER (Roy et al., 2010). Homology models were fitted into the reconstructed map of *Pf*80S using Chimera (Pettersen et al., 2004). Each protein was then subjected to a jiggle-fit and extensively rebuilt with sidechains placed into the map density using Coot v.0.8 (Emsley et al., 2010). The sequences of the *Pf*80S rRNAs were obtained from *PlasmoDB* (The Plasmodium Genome Database Collaborative, 2001) and aligned using Clustal Omega (Sievers et al., 2011) with the rRNA sequences extracted from the *Saccharomyces cerevisae (Sc)* 80S structure (PDB ID: 3U5B and 3U5D) (Ben-Shem et al., 2011). Conserved regions without insertions or deletions were extracted from the yeast structure, mutated and renumbered. These conserved sections were then connected by de novo building of RNA. The complete rRNA was then manually rebuilt in Coot to optimize the fit to density. Building was aided by secondary structure predictions downloaded from the Comparative RNA Website (Cannone et al., 2002).

The model was refined using REFMAC v.5.8, which was modified for structures determined by cryo-EM (Murshudov et al., 2011; Amunts et al., 2014). The *Pf*80S atomic model was refined as separate 60S and 40S subunits in the two maps that were obtained for either subunit in the focused refinements of the cryo-EM reconstructions. Structure factors for the (Fourier-space) refinement in REFMAC were obtained by cutting out sections of the corresponding maps with a 3 Å radius from the center of each atom in the model, and structure factor phases were not altered during refinement.

Throughout refinement, reference and secondary structure restraints were applied to the ribosomal proteins using the *Sc*80S structure as a reference model (Nicholls et al., 2012). Base pair and parallelization restraints obtained using LIBG were also applied throughout refinement (Amunts et al., 2014). The stereochemistry of the rRNA model was further improved using the ERRASER-PHENIX pipeline (Chou et al., 2013). Ramachandran restraints were not applied during refinement to preserve backbone dihedral angles for validation.

The R-factor and average overall Fourier shell correlation were monitored during refinement (Table 1) and the final model was validated using MolProbity (Chen et al., 2010). For cross-validation against over-fitting, we randomly displaced the atoms of our final model (with an RMSD of 0.5 Å) and performed a fully restrained refinement against a map that was reconstructed from only one of the two independent halves of the data that were used in our gold-standard FSC procedure. We then calculated FSC curves between the resulting model and the half-map against which it had been refined (FSC_work_), as well as the FSC curve between that model and the other half-map (FSC_test_). The observation that the FSC_work_ and FSC_test_ curves nearly overlap demonstrates the absence of overfitting of the model (Figure 1—figure supplement 1).

## Additional files

### Major datasets

The following datasets were generated:

WongW, BaiX-C, BrownA, FernandezIS, HanssenE, CondronC, TanYH, BaumJ, ScheresSHW, 2014, Plasmodium falciparum 80S ribosome bound to the anti-protozoan drug emetine, http://www.ebi.ac.uk/pdbe/entry/EMD-2660, Publicly available at Electron Microscopy Data Bank.

WongW, BaiX-C, BrownA, FernandezIS, HanssenE, CondronC, TanYH, BaumJ, ScheresSHW, 2014, Plasmodium falciparum 80S ribosome, http://www.ebi.ac.uk/pdbe/entry/EMD-2661, Publicly available at Electron Microscopy Data Bank.

WongW, BaiX-C, BrownA, FernandezIS, HanssenE, CondronC, TanYH, BaumJ, ScheresSHW, 2014, Cryo-EM structure of the Plasmodium falciparum 80S ribosome bound to the anti-protozoan drug emetine; large subunit, http://www.pdb.org/pdb/explore/explore.do?structureId=3J79, Publicly available at RCSB Protein Data Bank.

WongW, BaiX-C, BrownA, FernandezIS, HanssenE, CondronC, TanYH, BaumJ, ScheresSHW, 2014, Cryo-EM structure of the Plasmodium falciparum 80S ribosome bound to the anti-protozoan drug emetine; small subunit, http://www.pdb.org/pdb/explore/explore.do?structureId=3J7A, Publicly available at RCSB Protein Data Bank.

The following previously published datasets were used:

BaiX-C, FernadezIS, McMullanG, ScheresSHW, 2013, Ribosome structures at near-atomic resolution from thirty thousand cryo-EM particles, http://www.ebi.ac.uk/pdbe/entry/EMD-2275, Publicly available at Electron Microscopy Data Bank.

Ben-ShemA, Garreau de LoubresseN, MeinikovS, JennerL, YusupovG, YusupovM, 2011, The structure of the eukaryotic ribosome at 3.0 Å resolution, http://www.pdb.org/pdb/explore/explore.do?structureId=3U5B, Publicly available at RCSB Protein Data Bank.

Ben-ShemA, Garreau de LoubresseN, MeinikovS, JennerL, YusupovG, YusupovM, 2011, The structure of the eukaryotic ribosome at 3.0 Å resolution, http://www.pdb.org/pdb/explore/explore.do?structureId=3U5C, Publicly available at RCSB Protein Data Bank.

Ben-ShemA, Garreau de LoubresseN, MeinikovS, JennerL, YusupovG, YusupovM, 2011, The structure of the eukaryotic ribosome at 3.0 Å resolution, http://www.pdb.org/pdb/explore/explore.do?structureId=3U5D, Publicly available at RCSB Protein Data Bank.

Ben-ShemA, Garreau de LoubresseN, MeinikovS, JennerL, YusupovG, YusupovM, 2011, The structure of the eukaryotic ribosome at 3.0 Å resolution, http://www.pdb.org/pdb/explore/explore.do?structureId=3U5E, Publicly available at RCSB Protein Data Bank.

AngerAM, ArmacheJP, BerninghausenO, HabeckM, SubkleweM, WilsonDN, BeckmannR, 2013, Structure of the human 40S ribosomal proteins, http://www.pdb.org/pdb/explore/explore.do?structureId=3J3A, Publicly available at RCSB Protein Data Bank.

AngerAM, ArmacheJP, BerninghausenO, HabeckM, SubkleweM, WilsonDN, BeckmannR, 2013, Structure of the human 60S ribosomal proteins, http://www.pdb.org/pdb/explore/explore.do?structureId=3J3B, Publicly available at RCSB Protein Data Bank.

AngerAM, ArmacheJP, BerninghausenO, HabeckM, SubkleweM, WilsonDN, BeckmannR, 2013, Structure of the H. sapiens 40S rRNA and E-tRNA, http://www.pdb.org/pdb/explore/explore.do?structureId=3J3D, Publicly available at RCSB Protein Data Bank.

AngerAM, ArmacheJP, BerninghausenO, HabeckM, SubkleweM, WilsonDN, BeckmannR, 2013, Structure of the H. sapiens 60S rRNA, http://www.pdb.org/pdb/explore/explore.do?structureId=3J3F, Publicly available at RCSB Protein Data Bank.
