# Supplementary material for: Cryo-EM structure of the Plasmodium falciparum 80S ribosome bound to the anti-protozoan drug emetine
Source: eLife. 2014 Jun 9;3:e03080. doi: 10.7554/eLife.03080 (PMC4086275; doi:10.7554/eLife.03080)
Supplement: Related texts

# Article commentary: Decision letter

**DOI:** 10.7554/eLife.03080.018

**Affiliations:** Max Planck Institute of Biophysics, Germany

eLife posts the editorial decision letter and author response on a selection of the published articles (subject to the approval of the authors). An edited version of the letter sent to the authors after peer review is shown, indicating the substantive concerns or comments; minor concerns are not usually shown. Reviewers have the opportunity to discuss the decision before the letter is sent (see review process). Similarly, the author response typically shows only responses to the major concerns raised by the reviewers.

Thank you for sending your work entitled “Cryo-EM structure of the *Plasmodium falciparum* 80S ribosome bound to the anti-protozoan drug emetine” for consideration at *eLife*. Your article has been favorably evaluated by John Kuriyan (Senior editor) and 3 reviewers, one of whom, Werner Kühlbrandt, is a member of our Board of Reviewing Editors.

The Reviewing editor and the other reviewers discussed their comments before we reached this decision, and the Reviewing editor has assembled the following comments to help you prepare a revised submission.

All three reviewers agree that this is exciting, important, and innovative work that should be published in *eLife* as soon as possible. Two of the reviewers ask for a more detailed description and a more comprehensive comparison of this new structure with the few available structures of eukaryotic ribosomes, which is reasonable given the importance of the work. However, none of them have substantive concerns, so no major revisions are required.

Minor comments:

Reviewer #1:

1) For future reference it would be interesting to know how much material (in terms mg of purified 80S ribosomes) was available, and from roughly how much starting material (in terms of wet weight of cells) it was isolated.

2) For ease of comparison, please provide the concentration of the ribosome solution used for grid preparation in mg/ml, rather than only in terms of molarity.

3) Why is the resolution of the emetin complex significantly higher than that of the empty ribosome, even though the pixel size was larger? Is this fully explained by the larger number of particles averaged?

4) The tunnel exit should be labelled in Figure 2.

Reviewer #2:

1) The resolution is presented as 3.2A in the last paragraph of the Introduction and 3.4A in the first paragraph of the Results section. The authors need to correct or clarify these two apparently competing statements.

2) In the third paragraph of the Results section, a description of ESs refers to Figure 1C-D. Should this be Figure 2C-D?

3) In the fifth paragraph of the Results section: By “unmodified” are the authors meaning to indicate that this region is conserved?

4) Figure 3A. Is the protein shown in this panel eL41? If so, a label or mention in the legend should be added. Also, it is not clear which rRNA is being shown – is it 18S?

5) It would be helpful in Figure 3 and elsewhere to have an overall view of the 40S, 60S or 80S to orient the reader to the location of the detailed structures shown in the figures.

6) In the fifth paragraph of the Results: Should “Figure 3C” be Figure 3B? What is meant by the “P stalk”? Lastly, “Since the P and L1-stalks are required for coordinating movement of translation factors...” I am not aware of such a function for the L1 stalk.

7) In the Discussion section: What exactly is the “contour along one edge”? It was hard to tell from Figure 5B what was meant.

8) In general, it would be interesting to see more details of the comparison between this structure and the yeast 80S structure. For example, are they identical, apart from the phylogenetic variations? Are there any interesting conformational changes? What is the rmsd difference between residues in the conserved core? The paper is a bit disappointing in this regard, considering that this is the second structure for a complete eukaryotic ribosome.

*Reviewer #3*:

1) The authors should explicitly mention which compounds are currently used as front line drugs and cite a review.

2) Helices in the rRNA are labeled with abbreviations “H##”, also RNA nucleotides are named according to the convention “N##”, both of these abbreviations can cause confusion with single letter amino acid codes also used throughout - this should be changed to avoid ambiguity.

3) The structure of the *P. falciparium* ribosome is poorly described; it is critical to comment on how the basic architecture deviates (or not) from the crystal structures of previously solved eukaryotic ribosomes, Rabl et al 2011, Klinge et al 2011 and Ben-Shem et al 2011, based on which coordinates the *T. burucei* and the human ribosome has been modeled: otherwise talking about the core structure remains largely unreferenced (eL41 observed in Ben-Shem et al, polypeptide exit tunnel in Klinge et al, description of the architecture of the 40S subunit Rabl et al, etc...). The subsequent comparison with human ribosomes makes sense, as is.

4) Modifications to pactamycin led to compounds that maintain their antimalarial activity but with reduced toxicity. Can the authors comment on the possible mechanistic reasons for this observation?

5) Figure 2 – the orientation of the 80S is unusual, the authors should use an orientation that places the two subunits next to each other with the active site and the decoding center oriented up, nascent polypeptide exit tunnel down. This will facilitate comparison with other ribosome structures. Same applies in Figure 4.

6) The authors have used a currently unreleased version of Relion (1.3-beta) that includes some modifications. Typically, if modifications of previously published methods and software are used in a publication, the authors should describe it in detail and the program made available.

7) The 2D and 3D classification should be described in more detail (number of classes, particles per class).

8) The generation of masks for the focused refinement should be described in more detail.

# Reply: Author response

**DOI:** 10.7554/eLife.03080.019

Minor comments:

Reviewer #1:

*1) For future reference it would be interesting to know how much material (in terms mg of purified 80S ribosomes) was available, and from roughly how much starting material (in terms of wet weight of cells) it was isolated*.

We have now stated in the main text how many mg of ribosomes were yielded per gram of parasite.

*2) For ease of comparison, please provide the concentration of the ribosome solution used for grid preparation in mg/ml, rather than only in terms of molarity*.

We have now included the ribosome concentration in mg/ml.

3) Why is the resolution of the emetin complex significantly higher than that of the empty ribosome, even though the pixel size was larger? Is this fully explained by the larger number of particles averaged?

Pixel size is not the limiting factor in our reconstructions. The DQE of the Falcon-II camera is (nearly) flat out to ∼75% of Nyquist. Apparently, there is at least one other, as yet unidentified, bottleneck that restricts resolution before using a smaller pixel.

*4) The tunnel exit should be labelled in* *Figure 2*.

We have labelled the tunnel exit in Figure 2.

Reviewer #2:

*1) The resolution is presented as 3.2A in the last paragraph of the Introduction and 3.4A in the first paragraph of the Results section. The authors need to correct or clarify these two apparently competing statements*.

We have altered the text to clarify that the resolution of *Pf*80S in the absence of emetine was 3.4 Å, but reached 3.2 Å when emetine was present.

*2) In the third paragraph of the Results section, a description of ESs refers to* *Figure 1C-D**. Should this be* *Figure 2C-D**?*

We have altered the figure reference.

3) In the fifth paragraph of the Results section: By “unmodified” are the authors meaning to indicate that this region is conserved?

We have changed ‘unmodified’ to ‘conserved’.

*4)* *Figure 3A**. Is the protein shown in this panel eL41? If so, a label or mention in the legend should be added. Also, it is not clear which rRNA is being shown – is it 18S?*

The figure has been altered to improve the labelling.

*5) It would be helpful in* *Figure 3* *and elsewhere to have an overall view of the 40S, 60S or 80S to orient the reader to the location of the detailed structures shown in the figures*.

We have added overall views of *Pf*80S to clearly indicate the positions of the detailed features.

*6) In the fifth paragraph of the Results: Should “**Figure 3C**” be* *Figure 3B**? What is meant by the “P stalk”? Lastly, “Since the P and L1-stalks are required for coordinating movement of translation factors...” I am not aware of such a function for the L1 stalk*.

Yes! We have corrected the figure reference.

The P stalk is equivalent to the L7/L12 stalk in prokaryotes and is a lateral protuberance from the ribosome involved in the recruitment of translation factors. This is now stated in the text.

The L1 stalk is involved in coordinating movement of tRNAs during translocation (Trabuco, et al. (2010) J. Mol. Biol., 402(4):741-760). However, to avoid confusion with protein translation factors we have revised this sentence.

*7) In the Discussion section: What exactly is the “contour along one edge”? It was hard to tell from* *Figure 5B* *what was meant*.

In the emetine molecule there is an edge centred on the ethyl group and closest to the ribosome that could potentially be modified to improve the contacts with the ribosome. This is referred to as the ‘substitution contour’ in Figure 5B. We have modified the text to make this clearer.

*8) In general, it would be interesting to see more details of the comparison between this structure and the yeast 80S structure. For example, are they identical, apart from the phylogenetic variations? Are there any interesting conformational changes? What is the rmsd difference between residues in the conserved core? The paper is a bit disappointing in this regard, considering that this is the second structure for a complete eukaryotic ribosome*.

We have now added a substantial discussion of the comparison between the *Plasmodium* and yeast 80S structures focusing particularly on the composition of the two ribosomes and morphological differences in the quaternary structure. This includes a discussion of the absence of RACK1 and a suppressor protein and the inclusion of a tRNA bound at the E-site. We have calculated the RMSD for the conserved cores of the large and small subunits and for the head and body of the small subunit separately. We have also added additional differences between *Pf*80S and the human ribosome, notably the observation of an additional bridge between the platform of the 40S and eL8 in the large subunit.

Reviewer #3:

*1) The authors should explicitly mention which compounds are currently used as front line drugs and cite a review*.

We have added the identities of some of the most commonly used anti-malarials that are described in the reference ‘Grimberg BT, Mehlotra RK. Expanding the Antimalarial Drug Arsenal–Now, But How? Pharmaceuticals (Basel). 2011 May 1;4(5):681-712’.

*2) Helices in the rRNA are labeled with abbreviations “H##”, also RNA nucleotides are named according to the convention “N##”, both of these abbreviations can cause confusion with single letter amino acid codes also used throughout – this should be changed to avoid ambiguity*.

As recommended, we have altered the nomenclature used for labelling rRNA helices from H## to h##. We have also switched to using three-letter amino acid codes in the text.

*3) The structure of the* P. falciparium *ribosome is poorly described; it is critical to comment on how the basic architecture deviates (or not) from the crystal structures of previously solved eukaryotic ribosomes, Rabl et al 2011, Klinge et al 2011 and Ben-Shem et al 2011, based on which coordinates the* T. burucei *and the human ribosome has been modeled: otherwise talking about the core structure remains largely unreferenced (eL41 observed in Ben-Shem et al, polypeptide exit tunnel in Klinge et al, description of the architecture of the 40S subunit Rabl et al, etc...). The subsequent comparison with human ribosomes makes sense, as is*.

As described in the response to reviewer 2, we have now added a description of how *Pf*80S differs in architecture to the yeast 80S. The features of the core structure have now been referenced accordingly.

4) Modifications to pactamycin led to compounds that maintain their antimalarial activity but with reduced toxicity. Can the authors comment on the possible mechanistic reasons for this observation?

We have now commented possible mechanistic reasons for this observation in the Discussion.

*5)* *Figure 2* *– the orientation of the 80S is unusual, the authors should use an orientation that places the two subunits next to each other with the active site and the decoding center oriented up, nascent polypeptide exit tunnel down. This will facilitate comparison with other ribosome structures. Same applies in* *Figure 4*.

We have re-oriented the ribosome in all the relevant figures.

*6) The authors have used a currently unreleased version of Relion (1.3-beta) that includes some modifications. Typically, if modifications of previously published methods and software are used in a publication, the authors should describe it in detail and the program made available*.

The main features of the new algorithms are described in the Methods section. A more technical description will be presented in a dedicated paper that is currently in preparation. As we have done with all our software in the past, relion-1.3 will be made available as open-source to the community. The stable 1.3-release is planned in 1-2 months, pending updated documentation and final testing.

*7) The 2D and 3D classification should be described in more detail (number of classes, particles per class)*.

We have added to the Material and methods section a sentence describing how many rounds of 2D and 3D classification were performed for both data sets, and how many classes were used in each case.

*8) The generation of masks for the focused refinement should be described in more detail*.

An explanation of how the masks were generated has been added to the Materials and methods section.
